# Excitonic Physics in Emerging 2D Semiconductors: A First-Principles Many-Body Perspective from Photodetection to Solar Energy Conversion

**DOI:** 10.1021/acs.nanolett.6c03735

**Published:** 2026-10-12

**Authors:** Michele Re Fiorentin, Simone Grillo, Fulvio Paleari, Olivia Pulci, Daniele Varsano, Maurizia Palummo

**Affiliations:** † Department of Applied Science and Technology, 19032 Politecnico di Torino, corso Duca degli Abruzzi 24, 10129 Torino, Italy; ‡ Max Planck Institute for the Structure and Dynamics of Matter (MPSD) and Center for Free-Electron Laser Science (CFEL), 22761 Hamburg, Germany; ¶ Istituto Nanoscienze − CNR, S3, via G. Campi 213/A, 41125 Modena, Italy; § Dipartimento di Fisica and INFN, Università di Roma “Tor Vergata”, via della Ricerca Scientifica 1, 00133 Roma, Italy

**Keywords:** excitonic physics, 2D semiconductors, many-body perspective, polarization-sensitive photodetection, solar-energy conversion

## Abstract

Chemically and structurally diverse two-dimensional semiconductors exhibit a broad range of excitonic and optical phenomena that have direct implications for optoelectronic functionality. We examine these materials from a first-principles many-body perspective, emphasizing the connection between microscopic excitonic structure and optoelectronic functionality. Reduced symmetry and orbital character produce polarization-selective absorption, while dimensionality, dielectric screening, and exciton fine structure govern binding energies and radiative behavior. In layered systems, interlayer electrostatics and type-II band alignment promote charge separation, whereas exciton–phonon coupling activates indirect optical transitions and shapes temperature-dependent spectra. These mechanisms establish common design principles for excitonic engineering, whereby the dielectric environment, crystal geometry, lattice dynamics, and stacking are used to control exciton properties and tailor optical functionality. This perspective moves beyond description toward the *ab initio* prediction and design of emerging 2D materials for targeted optoelectronic applications, including polarization-sensitive photodetection and ultrathin solar-energy conversion.

## Graphical abstract

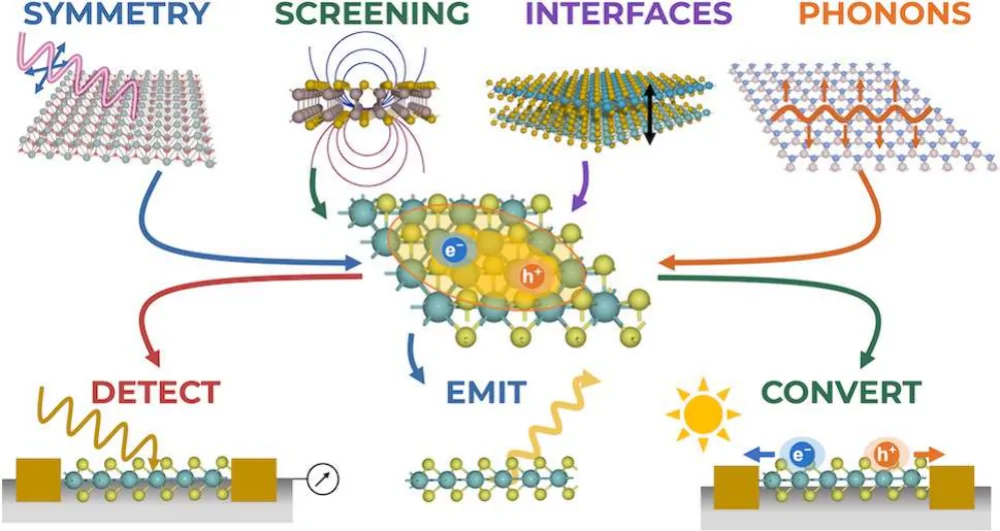

Two-dimensional (2D) semiconductors provide an exceptional platform in which excitonic effects dominate light–matter interaction. In atomically thin layers, charge carriers are confined within the plane, while electric fields extend into the surrounding three-dimensional environment^1,2^ Confinement weakens dielectric screening and enhances electron–hole attraction, producing strongly bound excitons that govern optical absorption and emission even at room temperature.^3–5^ As a consequence, optical spectra in 2D materials are governed not only by the underlying electronic band structure, but also by the spatial structure, dispersion, localization, symmetry, and dynamics of excitons, making their controlled manipulation a central strategy for tailoring optical functionality and designing optoelectronic devices.^6^ Although early developments focused largely on transition metal dichalcogenides,^7,8^ a growing family of emerging 2D semiconductors is now considerably expanding the range of possibilities for excitonic engineering. Ultrawide-band-gap nitrides,^9^ low-symmetry monochalcogenides,^10^ carbon-based layered networks,^11^ and recently synthesized MoSi_2_X_4_ compounds^12^ provide access to diverse electronic structures, dielectric responses, and lattice symmetries. These materials not only broaden the accessible spectral window from the ultraviolet to the near-infrared but also enable new regimes of anisotropic, environment-sensitive, and long-lived excitonic behavior, opening opportunities for Coulomb engineering through dielectric screening.^13,14^

Across these platforms, excitonic properties are governed primarily by a small set of microscopic design parameters. Reduced crystal symmetry frequently breaks in-plane rotational invariance, leading to anisotropic optical selection rules and polarization-dependent absorption.^15^ At the same time, exciton–phonon coupling governs exciton formation, thermalization, and linewidth broadening,^16^ while enabling phonon-assisted recombination channels and access to momentum-forbidden excitonic states.^17,18^ Finally, interlayer electrostatics in van der Waals heterostructures (vdWHs) provides a powerful route to manipulate exciton dissociation and charge transfer,^19,20^ allowing for long-lived interlayer excitons and efficient carrier separation.

Rather than reviewing excitonic phenomena material by material, we organize this Mini-Review around these microscopic mechanisms. We discuss how symmetry, interlayer electrostatics, and lattice dynamics govern excitonic physics in emerging 2D semiconductors and how they provide complementary routes to engineer polarization-selective absorption, radiative recombination, and charge separation for photodetection and solar energy conversion in ultrathin optoelectronic platforms.

## Ab Initio Many-Body Framework for Excitonic and Optical Properties

Predictive modeling of optical spectra in 2D semiconductors typically follows a many-body perturbation theory workflow, in which density functional theory (DFT) within the Kohn–Sham formalism provides the ground-state electronic structure used as the starting point for quasiparticle and excitonic calculations.^21,22^ Quasiparticle excitation energies are then obtained within the *GW* approximation,^23,24^ where the electronic nonlocal and frequency-dependent self-energy Σ = i*GW* replaces the DFT exchange–correlation potential. In 2D materials, the dielectric screening *W* is strongly reduced and intrinsically nonlocal, making its accurate evaluation critical.^25,26^

Building upon the *GW* quasiparticle electronic structure, optical excitations are described by the Bethe–Salpeter equation (BSE), whose two-body Hamiltonian accounts for the correlated electron–hole interaction through screened direct and bare exchange terms.^27,28^ The diagonalization of the BSE Hamiltonian yields the exciton energies *E*_*S*_ and the eigenvectors $A_{vck}^{S}$ , which determine the optical response of the material. Depending on spin, momentum, and symmetry selection rules, the resulting excitons may be optically bright or dark.^29,30^ Moreover, diagonalizing the BSE Hamiltonian at different transferred momenta **Q** gives access to exciton dispersion *E*_*S*_(**Q**), which can be probed by EELS/IXS spectroscopy.^31,32^ Beyond excitation energies, the BSE eigenvectors define the correlated electron–hole wave function,

1 $$\Psi_{S}\left(r_{e},r_{h}\right)=\underset{vck}{\sum }A_{vck}^{S}\psi_{ck}\left(r_{e}\right)\psi_{vk}^{*}\left(r_{h}\right)$$

which reveals the spatial extent, anisotropy, electron–hole correlation, and, in layered systems, the intra- or interlayer character of the exciton.

In the case of 2D materials, the absorption spectrum is proportional to the imaginary part of the 2D polarizability:^2^

2 $$\alpha_{2D}\left(\omega \right)=\frac{2}{A}\underset{S}{\sum }\frac{{\left|e\cdot d_{S}\right|}^{2}}{E_{S}-\omega -i\eta }$$

where *A* represents the unit-cell area of the material, **d**_*S*_ = $\frac{1}{N_{k}}\underset{cvk}{\sum }A_{cvk}^{S}\left\langle ck\left|r\right|vk\right\rangle$ is the exciton transition dipole obtained from the BSE eigenvectors, and **e** is the light polarization vector. The magnitude and orientation of **d**_*S*_ encode the crystal symmetry and orbital character of the exciton, thus determining polarization-dependent optical selection rules. Consequently, crystal symmetry, anisotropic orbital textures, and stacking-induced symmetry breaking, directly reshape the exciton transition dipoles, redistributing the oscillator strength among different polarization directions. These microscopic changes ultimately determine polarization-selective optical responses and provide a direct route to engineering anisotropic optoelectronic functionality.

Beyond linear response, excitonic correlations can strongly modify nonlinear optical processes that arise at sufficiently strong fields, when the optical response is no longer proportional to the incident field. Real-time BSE and nonequilibrium *GW* provide a natural framework for these strong-field phenomena, such as high-harmonic generation, while coherent spectroscopy probes exciton dephasing and coupling.^33–35^

More generally, nonequilibrium Green’s function approaches simulate transient spectra and pump–probe dynamics, including the evolution from coherent polarization to incoherent exciton populations.^33,36^ Such capabilities are increasingly available in public first-principles codes, with new numerical implementations following increasingly sophisticated theoretical developments.^37–39^

Solving the BSE also provides intrinsic exciton radiative lifetimes from the excitonic transition dipole moments. For a bright exciton state *S* with energy *E*_*S*_, the intrinsic radiative decay rate in a 2D material can be written as^40^

3 $$\gamma_{S}=\frac{2\pi e^{2}E_{S}}{\hbar^{2}c^{2}A}{\left|e\cdot d_{S}\right|}^{2}$$

Radiative recombination is restricted to excitons inside the light cone, namely those with center-of-mass momentum |**Q**| ≤ *E*_*S*_/*ℏc*. At finite temperature, the exciton population spreads over larger-momentum states that are optically inactive, reducing the fraction of excitons able to emit and leading, for a thermalized parabolic exciton dispersion in 2D, to the characteristic linear scaling $\left\langle \tau_{S}\right\rangle \propto \gamma_{S}^{-1}T$ .

In many materials, exciton dynamics is strongly influenced by lattice vibrations. Exciton–phonon coupling governs exciton thermalization, line width broadening, and relaxation between bright and dark states, while enabling phonon-assisted optical transitions in indirect-gap systems. Within the *GW*-BSE framework, this interaction is described by the exciton–phonon matrix elements $G_{nm\nu }\left(Q,q\right)$ , which couple an initial exciton *S*_*n*_ to a final exciton *S*_*m*_ through the absorption or emission of a phonon mode (ν, **q**). The coupling is obtained as a weighted superposition of the underlying electron–phonon matrix elements, where the excitonic wave functions determine how the electron and hole contributions combine. Consequently, the exciton–phonon interaction reflects both the momentum dependence of the electron–phonon coupling and the internal structure of the exciton.^41,42^

From these matrix elements, one can compute phonon-assisted absorption and emission spectra,^17,43–45^ exciton relaxation lifetimes,^46,47^ and resonant Raman spectra.^48^ For example, the phonon-assisted satellite contribution can be described as a second-order process involving one photon and one phonon and written as^43^

4 $$I_{sat}\left(\omega \right)=\frac{1}{3N_{q}}\underset{\begin{array}{c} \lambda ,n,\nu ,q\\ s=\pm 1 \end{array}}{\sum }\frac{1}{E_{nq}-s\Omega_{\nu q}}{\left|\underset{m}{\sum }\frac{e_{\lambda }\cdot d_{m}^{*}G_{nm\nu }^{*}\left(q\right)}{E_{m0}-E_{nq}+s\Omega_{\nu q}+i\eta }\right|}^{2}\times N_{nq}^{exc}\left[\frac{1+s}{2}+n_{\nu q}\right]\frac{1}{\omega -\left(E_{nq}-s\Omega_{\nu q}\right)+i\eta }$$

where *n* labels the initial finite-momentum exciton *S*_*n*_ with center-of-mass momentum **q** and *m* labels the intermediate virtual bright exciton *S*_*m*_ at zero momentum. Furthermore, $N_{nq}^{exc}$ and *n*_ν**q**_ denote the exciton and phonon occupation factors, respectively, and Ω_ν**q**_ is the phonon energy. The index *s* = +1 corresponds to phonon emission, whereas *s* = −1 corresponds to phonon absorption. Finally, the sum over **e**_λ_, together with the prefactor 1/3, accounts for the average over the polarization directions of the emitted photon. Phonon-assisted optical satellites have the energy of the momentum-dark *S* *_n_* exciton plus or minus the phonon energy. Equation 4 shows that phonon-assisted emission proceeds through optically bright intermediate excitons *S*_*m*_ at **Q** = 0, requiring both a finite optical dipole moment (**d**_*m*_ ≠ 0) and a nonvanishing exciton–phonon coupling $\left(G_{nm\nu }\neq 0\right)$ . This provides the microscopic basis for the phonon replicas discussed later.

Despite their predictive power, quantitative *GW*-BSE calculations remain computationally demanding and are highly sensitive to parameters such as k-point sampling, dielectric cutoffs, empty states, and vacuum separation. In 2D systems, long-range screening requires dense momentum grids due to the Coulomb truncation,^49^ creating a major computational bottleneck. Recent algorithmic developments, such as accelerated convergence schemes^26^ and double-**k**-grid sampling strategies,^50^ substantially reduce these costs. Furthermore, exploiting modern GPU-accelerated hybrid architectures provides dramatic speedups for *GW*-BSE codes, enabling the study of significantly larger systems than those feasible on traditional machines.

Altogether, the *GW*-BSE framework provides a unified microscopic description linking the crystal symmetry, dielectric screening, and lattice dynamics to excitonic properties. This quantitative connection between the atomic structure and the optical response forms the basis for the design principles discussed in the following sections.

### Reduced Symmetry and Optical Anisotropy

Within this many-body framework, symmetry dictates both the structure and optical activity of excitons.

In contrast to high-symmetry hexagonal systems, low-symmetry 2D materials, such as black phosphorus and group IV monochalcogenides, possess inequivalent in-plane crystallographic directions. This inherent anisotropy reshapes the orbital character, effective masses, and dipole selection rules near the band edges, resulting in strongly directional absorption and emission. In phosphorene, for instance, the puckered structure leads to markedly different electronic dispersions along armchair and zigzag directions, resulting in pronounced linear dichroism that is further enhanced by excitonic effects.^51^ This microscopic picture is directly verified experimentally through polarization-resolved Raman, photoluminescence, and absorption spectroscopy, which establish a one-to-one correspondence between the crystal orientation and optical response in layered monochalcogenides.^10^

Beyond a single structural phase, polymorphism introduces an additional degree of control over anisotropic excitonic properties. In tellurene, different crystalline phases exhibit distinct excitonic spectra and large optical absorbance, extending across the visible–near-infrared range, together with pronounced in-plane anisotropy.^52^ The coexistence of large absorbance and pronounced anisotropy makes tellurene particularly attractive for polarization-sensitive photodetection across the visible and near-infrared spectral range. This highlights how structural variability at the atomic scale can be used to tune both the magnitude and spectral window of anisotropic optical responses, enabling material-specific targeting of functional wavelength ranges.

A more subtle and less common situation arises when the preferred polarization depends on photon energy, due to the coexistence of excitonic transitions with different orbital characters. This is illustrated in Figure 1b for monolayer SnO, where different excitonic peaks exhibit opposite polarization dependence.^15^ As shown in Figure 1c, the absorption maxima rotate by 90° between the main resonances at different light frequencies, and Figure 1a reveals a corresponding sign change of the degree of linear polarization (DLP) across the spectrum. A separate *GW*-BSE study predicts a similarly anisotropic SnO response near 4.25 eV, close to the 4.33 eV resonance discussed here.^53^ Monolayer SnO has also been experimentally realized and photoluminescence reported,^54^ although polarization-resolved spectroscopy of the predicted dichroism inversion remains lacking. Microscopically, the inversion reflects excitonic states from inequivalent Brillouin zone regions with different p_*x*_/p_*y*_ orbital character and therefore polarization-dependent optical matrix elements. In this sense, reduced symmetry acts as a functional design principle: by controlling lattice symmetry and orbital character, one can directly engineer the polarization selectivity and directional response of excitonic transitions. This provides a direct route to polarization-selective photodetection, where the absorption of light can be modulated by crystal orientation without the need for external optical elements. Moreover, frequency-dependent dichroism enables spectral-polarization selectivity, where different photon energies can be preferentially absorbed along orthogonal directions, offering a mechanism for multichannel photodetection within a single material.

**Figure 1:**
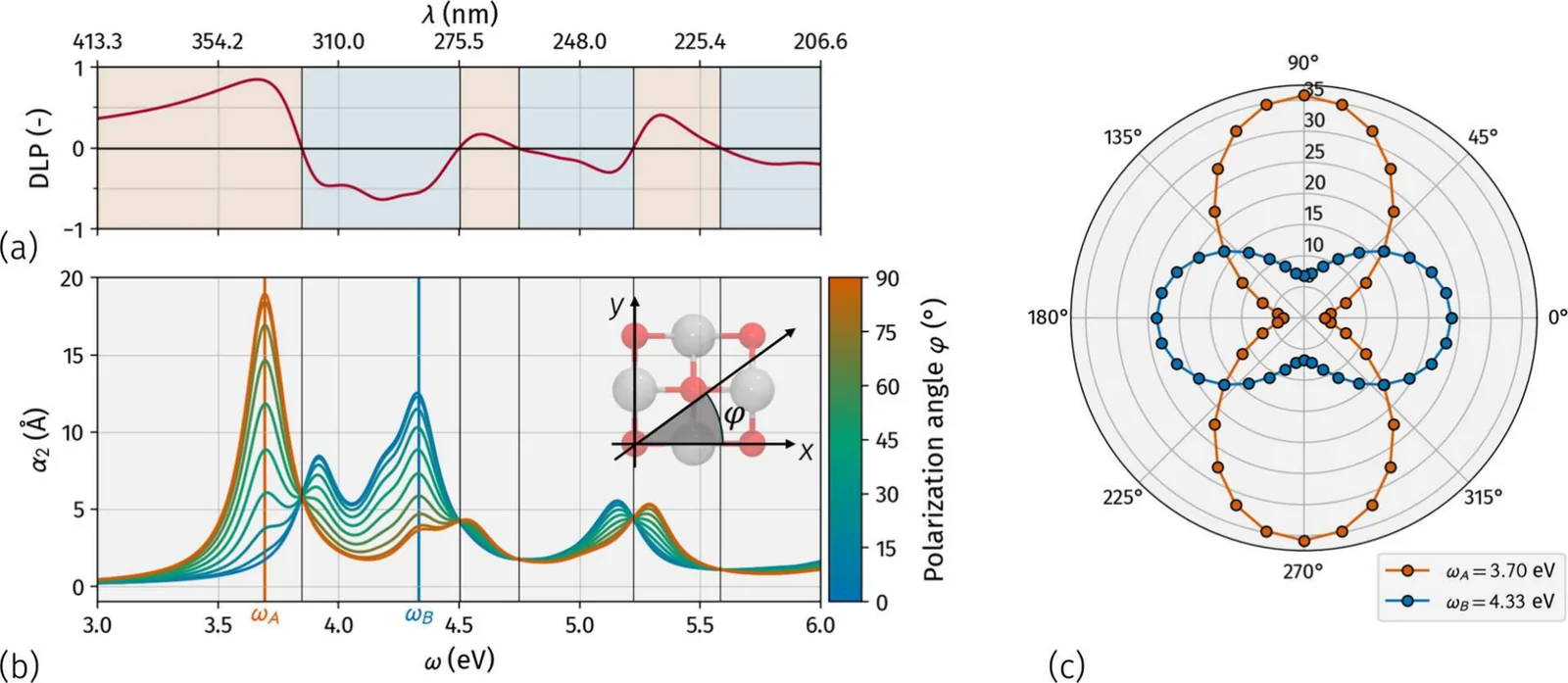
Linear dichroism inversion with photon energy in monolayer SnO. (a) Degree of linear polarization (DLP). (b) Absorption spectra for different in-plane light polarization angles φ. (c) Polar plot of absorption at lower (ω_*A*_) and higher (ω_*B*_) frequencies. Adapted from ref 15. Copyright 2025 American Chemical Society.

Finally, symmetry can be further engineered through stacking in 2D/layered materials.^55,56^ In hexagonal boron nitride, different stacking sequences (polytypes) modify optical selection rules and activate or suppress specific excitonic transitions, providing an additional symmetry axis to control the optical activity.^43^

Taken together, these examples illustrate a general design strategy: by engineering the crystal symmetry through lattice geometry, polymorphism, or stacking, one can tailor excitonic selection rules and polarization-dependent optical responses without modifying the underlying chemistry of the material.

### Dimensionality, Excitonic Binding, and Radiative Lifetimes

Dimensionality, dielectric screening, and electronic structure jointly determine the fundamental properties of excitons in 2D semiconductors, most notably their binding energies and radiative lifetimes. Reduced dimensionality enhances the Coulomb interaction and leads to large exciton binding energies; however, binding energy alone does not determine optical activity. Radiative recombination rates in materials are instead governed by the exciton fine structure, including the relative ordering of bright and dark states, the distribution of oscillator strengths, and the available relaxation pathways in the momentum space. Strongly bound excitons in 2D materials can be either optically inactive or highly emissive, depending on how the crystal geometry, symmetry, and band topology shape the lowest-energy excitations. These quantities directly control the emission efficiency, carrier lifetime, and energy conversion processes in optoelectronic devices.

2D graphitic carbon nitrides (gC_3_N_4_) provide a clear example of the connection between material geometry and excitonic properties.^11^ As shown in Figure 2a,b, the triazine (gC_3_N_4_-t) and heptazine (gC_3_N_4_-h) phases both exhibit pronounced excitonic effects, with absorption spectra dominated by discrete bound states well below the independent-quasiparticle continuum. The exciton fine structure is dominated by a manifold of dark low-energy states, so that the excitonic ground state is optically inactive and the oscillator strength is redistributed toward higher-energy bright excitons. The two polymorphs illustrate how subtle structural changes can reshape the exciton landscape: the heptazine phase hosts a denser manifold of dark states (marked *H*_0*i*_ in Figure 2b), resulting in effective radiative lifetimes reaching hundreds of nanoseconds, whereas the triazine phase remains in the nanosecond regime, as shown in the lower panel of Figure 2a. Recent *GW*-BSE calculations likewise find structure-dependent dark-exciton lifetimes from nanoseconds to micro/milliseconds,^57,58^ while photoluminescence and ultrafast measurements confirm strong excitonic effects and interfacial charge separation in gC_3_N_4_.^59–61^ Such long-lived states favor charge extraction in photocatalytic and energy-conversion applications.

**Figure 2:**
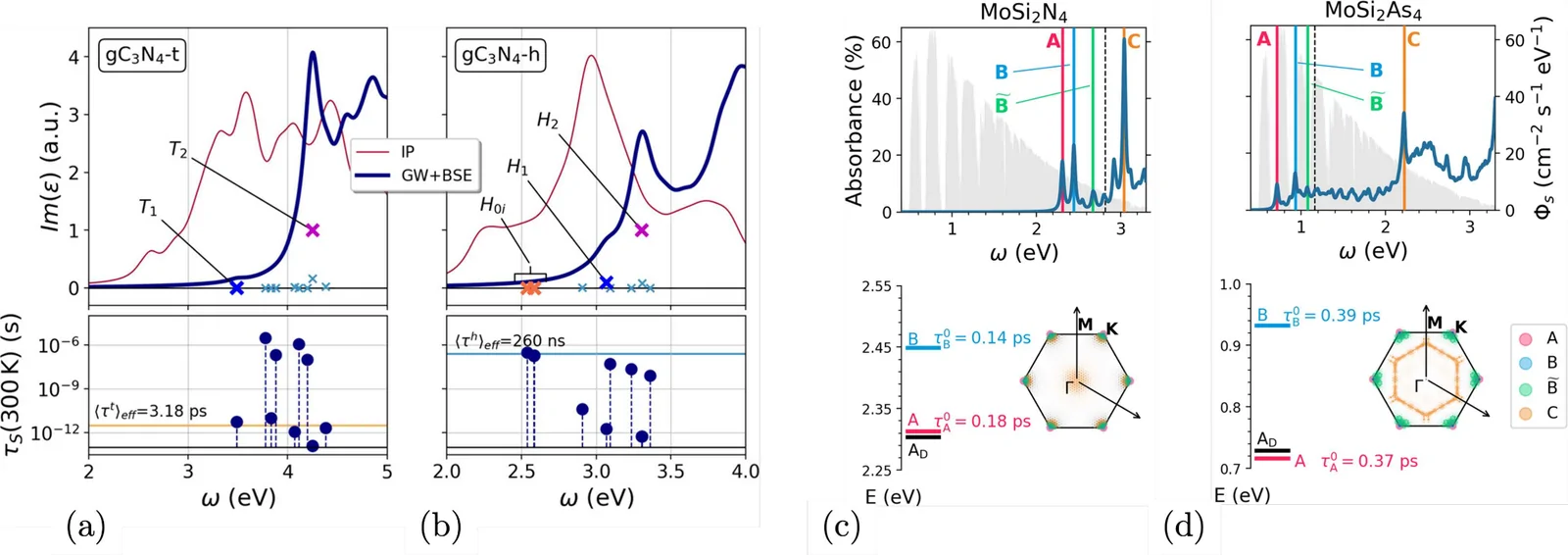
(a, b) Absorption spectra and radiative lifetimes in 2D materials. Optical absorption and exciton radiative lifetimes for the gC_3_N_4_ polymorphs triazine in (a) and heptazine in (b). Adapted from ref 11. CC BY 4.0. (c, d) Absorption, exciton energy ordering, radiative lifetimes, and exciton weights in the Brillouin zone for MoSi_2_N_4_ in (c) and MoSi_2_As_4_ in (d). Adapted from ref 12. CC BY 4.0.

A distinct excitonic regime is realized in the MoSi_2_X_4_ family, where the dielectric outer layers modify both the strength of electron–hole interactions and the fine structure of the exciton.^12^ This leads to smaller exciton binding energies in comparison to more strongly confined systems, while preserving a sizable excitonic character. A separate *GW*-BSE study^62^ reports *E*_b_ = 0.63–0.78 eV for the A exciton, bracketing the 0.68 eV value of ref 12. Most importantly, the ordering of excitonic states evolves along group-V series: while the nitride retains a dark low-energy exciton (Figure 2c), the arsenide exhibits a low-energy bright exciton (Figure 2d). Consequently, MoSi_2_As_4_ is intrinsically emissive, whereas in MoSi_2_N_4_ exciton relaxation into dark states suppresses radiative recombination. Experimentally, the MoSi_2_N_4_ A/B excitons near 2.2/2.35 eV agree closely with *GW*-BSE predictions,^63,64^ although the dark–bright ordering remains unverified. MoSi_2_As_4_ has not yet been experimentally realized. This microscopic picture is reflected in the lower panels of Figure 2c,d, where the exciton energy ordering, valley character, and intrinsic radiative lifetimes evolve consistently throughout the series. The inversion between dark and bright excitonic ground states therefore provides a direct materials-design strategy, allowing radiative efficiency to be tuned through composition without fundamentally altering the crystal structure.

A complementary limit is represented by group-III nitride monolayers, where strong exciton binding coexists with intrinsically bright lowest-energy excitons and ultrafast radiative recombination. First-principles studies of honeycomb BN, AlN, GaN, InN, and TlN^9^ show that chemical composition strongly tunes the quasiparticle electronic structure: the gap decreases from the deep-UV range in BN and AlN to the visible/near-infrared range in InN (Figure 3a), while TlN approaches a nearly gapless limit.^65^ BN and AlN are indirect semiconductors, whereas GaN and InN exhibit direct gaps at Γ. Graphene-encapsulated 2D GaN has been experimentally realized with a direct gap near 5 eV, although its structure differs from the ideal freestanding monolayer.^66^ Alloying further extends this tunability, with In_*x*_Ga_1–*x*_N and In_*x*_Tl_1–*x*_N enabling composition-dependent band offsets and type-I or type-II heterostructures, relevant for radiative recombination or charge separation (see Figure 3c).

**Figure 3:**
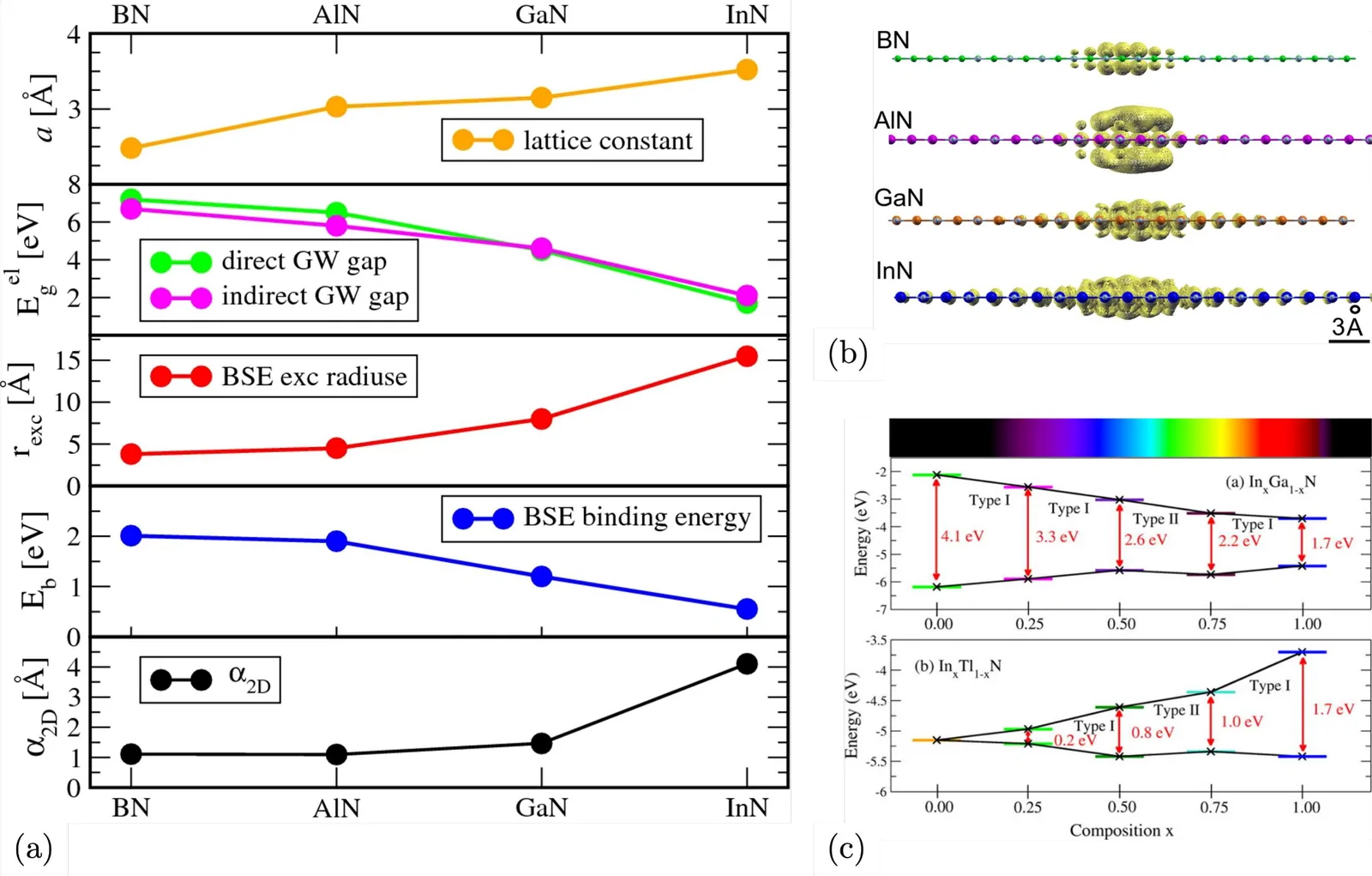
Trends in Group III-Nitrides. (a) Lattice constant, direct and indirect quasiparticle gaps, exciton radius, exciton binding energy and 2D static polarizability for BN, AlN, GaN, and InN. (b) Exciton wave functions for the 2D nitrides. Adapted from ref 9. CC BY 4.0. (c) Direct band gap and band extrema with respect to the vacuum level of In_*x*_Ga_1–*x*_N (upper panel) and In_*x*_Tl_1–*x*_N (lower panel). Adapted with permission from ref 65. Copyright 2017 AIP Publishing.

When quasiparticle and electron–hole interaction effects are included, BN, AlN, GaN, and InN monolayers display large bound excitons, with binding energies decreasing from about 2.0 eV in BN to 0.6 eV in InN, while the exciton radius increases from 3.8 to 15.5 Å along the same series, as shown in Figure 3b. Additional many-body calculations give *E*_b_ = 1.23–1.27 eV for monolayer GaN, consistent with the 1.2 eV value of ref 65, and support composition-dependent excitonic tuning in III-nitride alloys.^67,68^ As a consequence, zero-momentum radiative lifetimes are only tens of femtoseconds, while thermally averaged room-temperature lifetimes range from tens of picoseconds in BN, AlN, and GaN to a few hundred picoseconds in InN.^9^ InN nanosheets further illustrate the role of layer number: going from monolayer to AA′ bilayer InN reduces the direct *GW* gap from 1.72 to 0.86 eV and the BSE optical onset from 1.17 to 0.42 eV, while the exciton binding energy decreases from 0.55 to 0.44 eV, because of weaker confinement and enhanced screening.^69^ Thus, reduced dimensionality in 2D nitrides produces bright, strongly bound excitons with rapid radiative decay, where symmetry, band-edge character, and optical matrix elements favor emission.

These examples define distinct excitonic regimes that can be accessed through different microscopic design parameters. Dimensionality and dielectric screening primarily determine the strength of electron–hole binding, whereas crystal geometry, composition, and the dielectric environment reshape the exciton fine structure, enabling transitions between dark- and bright-ground-state regimes with direct consequences for light emission, carrier extraction, and energy conversion.

### Interfaces and Heterostructures for Charge Separation and Photogeneration

Interfaces and heterostructures provide the most effective strategy for overcoming one of the central challenges of excitonic photovoltaics: the separation of strongly bound electron–hole pairs into free carriers before radiative recombination occurs.^11,70–72^ vdWHs offer a uniquely flexible platform for this purpose, since electronically dissimilar layers can be vertically assembled without lattice-matching constraints, allowing independent engineering of band alignment, interlayer hybridization, strain, and built-in electric fields.^73,74^ Type-II band alignment is especially relevant for photovoltaic applications, as band discontinuities drive photogenerated carriers toward opposite layers, promoting ultrafast interlayer charge transfer and the formation of spatially indirect excitons with extended lifetimes.^11,20,71,75,76^ The resulting spatial separation of electrons and holes reduces their wave function overlap, suppressing radiative recombination, while promoting charge extraction. At the same time, efficient photogeneration requires strong absorption across the solar spectrum, so that enhanced charge separation does not come at the expense of light harvesting. Bernardi et al. quantified this balance for a 1 nm-thick MoS_2_/WS_2_ vdWH, predicting a maximum short-circuit current density of *J*_sc_^max^ ≈ 3.5 mA/cm^2^, approximately 1 order of magnitude larger than that of GaAs or Si at the same thickness.^77^ This benchmark highlights that maximizing photovoltaic performance requires simultaneously optimizing light absorption and interfacial charge separation.^20^ Two recent *GW*-BSE studies have identified strong candidates in this direction. Bastonero et al.^78^ showed that band nesting in monolayer 1T-PdS_2_ produces absorbance peaks approaching 55%. When combined with 1T-PtS_2_ in a type-II vdWH, the predicted short-circuit current reaches *J*_sc_^max^ ≈ 7.2 mA/cm^–2^, approximately twice that of MoS_2_/WS_2_. The indirect interlayer exciton further suppresses radiative recombination, extending carrier diffusion lengths beyond 20 μm.

Tellurene provides a complementary route by combining a thickness-tunable electronic structure with high carrier mobility, environmental stability, and scalable synthesis.^79–81^ Its α, β, and γ polymorphs, whose structures are shown in Figure 4a, exhibit optical absorbances reaching ∼60% in the near-infrared and visible range, with *J*_sc_^max^ values of 8.4, 2.2, and 7.1 mA/cm^2^ respectively (see Figure 4b) and room-temperature-stable excitons with binding energies of 0.28–0.50 eV.^52^ A separate many-body study of α-tellurene^82^ reports *E*_b_ = 0.31 eV and *J*_sc_^max^ = 6.7 mA/cm^2^, consistent with the corresponding values discussed above. The orthorhombic phases further display marked in-plane optical anisotropy that could introduce polarization selectivity at photogenerating interfaces. Experiments confirm strong near-infrared absorption and in-plane anisotropy in few-layer tellurene and the realization of monolayer α-Te,^83–86^ although phase-resolved absolute absorbance and photovoltaic measurements remain lacking. A systematic *GW* band alignment survey against several 2D materials^65,69,87^ reveals favorable type-II offsets across numerous combinations, see Figure 4c. The β-Te-based junctions are predicted to reach power conversion efficiencies of 11–19%, as shown in Figure 4d, with the β-Te/γ-Te homojunction estimated at ∼19%, approaching the crystalline-Si technology at a thickness of only ∼1 nm. Related calculations of type-II Te/TMD junctions predict efficiencies of 20–22.5%, consistent with the photovoltaic-performance range of tellurene-based heterostructures.^88^ These efficiencies are ideal upper bounds. Real devices also face carrier-collection, recombination, contact, band-alignment,^89^ and stability losses.^90^ Nonetheless, these results establish tellurene as both a promising, exceptional ultrathin absorber and a versatile building block for polymorph-engineered van der Waals charge-separating interfaces.^52^

**Figure 4:**
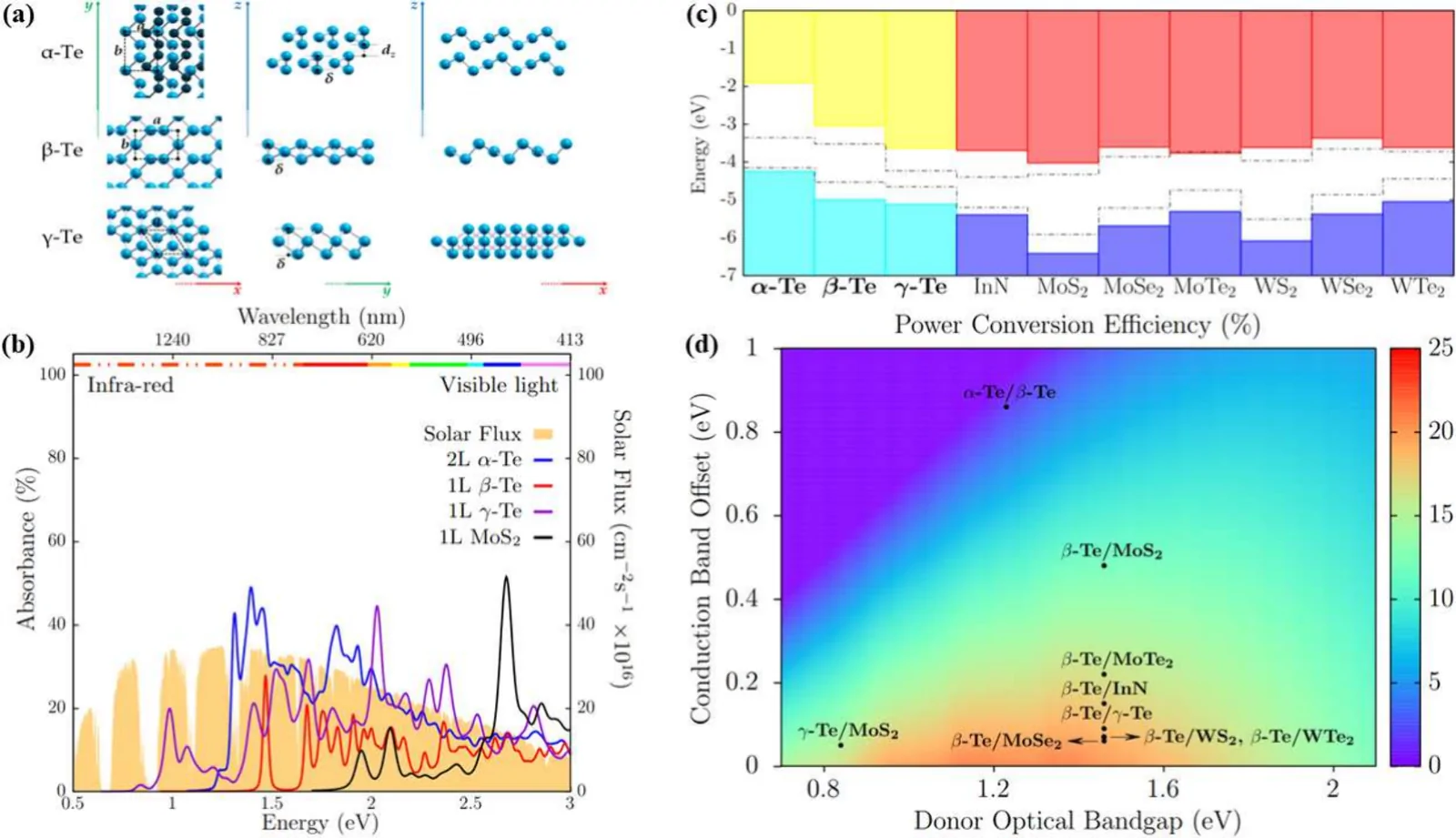
Optoelectronic properties of tellurene polymorphs. (a) Optimized geometric structures of 2L α-Te, 1L β-Te, and 1L γ-Te. (b) Comparison of the optical absorption spectra of 2L α-Te, 1L β-Te, and 1L γ-Te with MoS_2_ (black). (c) Comparison of the band-offset alignment between 2L α-Te, 1L β-Te, and 1L γ-Te and other monolayer 2D semiconducting materials calculated at the at *GW* level. Black dashed lines show the relative alignment from DFT calculations. (d) Heatmap of the power conversion efficiency plotted as a function of the donor optical band gap and of the conduction band offsets. The black dots refer to the efficiency of the heterostructures shown in (c). Adapted with permission from ref 52. Copyright 2024 Wiley-VCH.

Unlike isolated monolayers, where optical absorption and carrier recombination are largely governed by the same excitonic states, interfaces enable these processes to be engineered independently. By combining a suitable band alignment with strong light absorption and controlled interlayer exciton formation, vdWHs provide a versatile platform for optimizing ultrathin photodetectors and photovoltaic devices.

### Exciton–Phonon Coupling and Temperature-Dependent Optics

Exciton–phonon coupling is a key ingredient of the optical response of low-dimensional semiconductors, governing temperature-dependent line widths, exciton relaxation, and access to momentum-forbidden dark excitons through phonon-mediated scattering.^5^ Moreover, many multilayer and heterostructured 2D systems possess an indirect fundamental gap, making phonon-assisted processes the dominant radiative recombination channel. A quantitative description of exciton–phonon interactions is therefore essential for interpreting optical spectra and designing indirect optoelectronic materials. The microscopic picture begins with the exciton dispersion, obtained by diagonalizing the BSE at finite exciton momentum. Figure 5a–d reports the exciton band structure of bilayer WSe_2_,^91^ which exhibits a rich multivalley excitonic landscape, due to the presence of several indirect electronic transition at different momentum transfer in the same energy range. The optoelectronic response is primarily determined by the momentum, spin–orbital character, real-space localization (intralayer versus interlayer), and relative energies of the excitonic states. These quantities are highly tunable through stacking, strain, external electric fields, chemical composition, doping, and dielectric environment. Figure 5a,b illustrates this behavior for bilayer WSe_2_. The AA′ and AB stackings present reversed intralayer (DX_IP_) versus interlayer (DX_IL_) exciton energy order at the optical limit **Q** = Γ, and the excitonic ground state is located at a different finite-**Q** valley, which has consequences for exciton relaxation dynamics. Figure 5c,d shows that electric fields selectively shift the interlayer excitons, modifying their ordering with respect to intralayer states in a valley-dependent manner.

**Figure 5:**
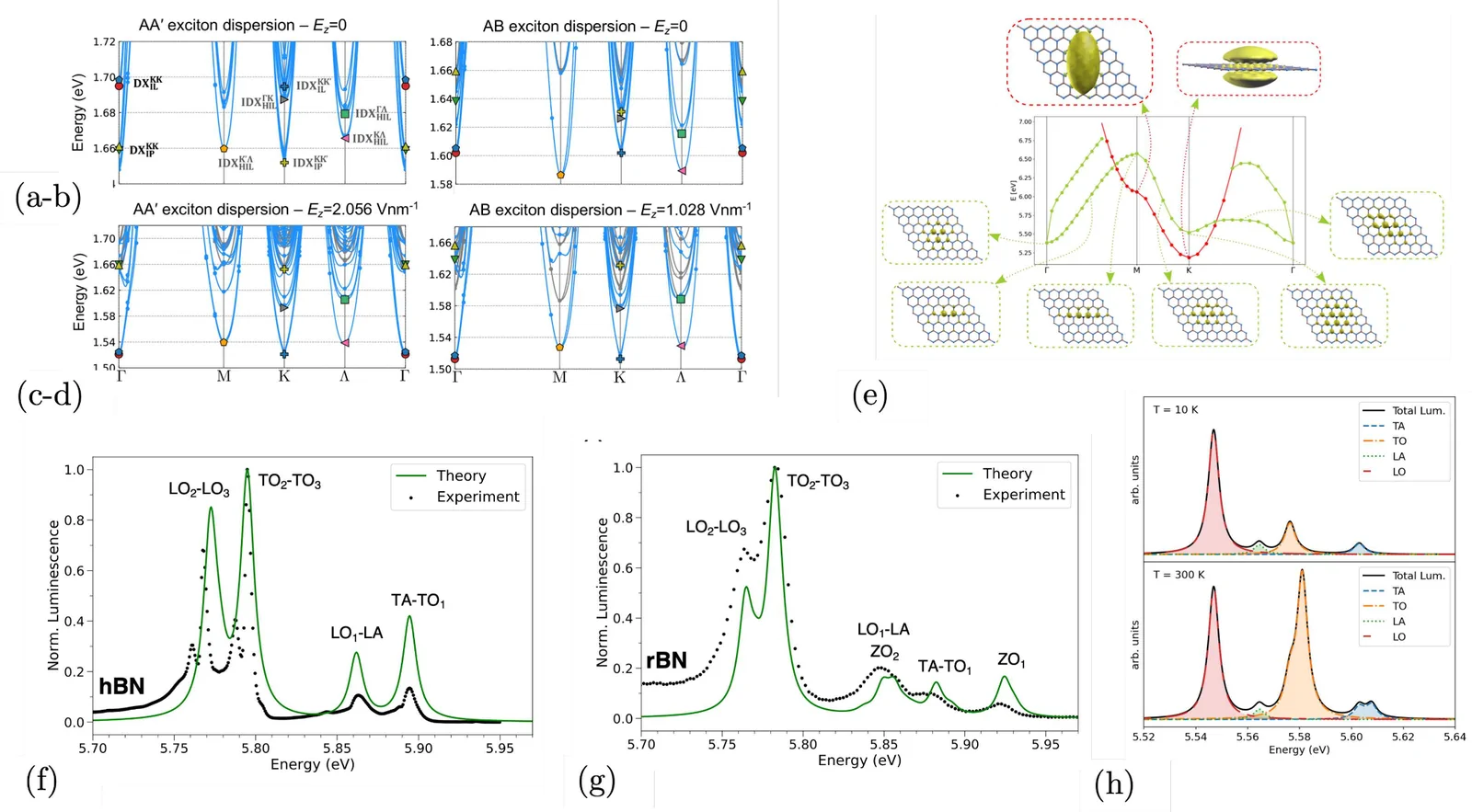
Exciton–phonon interactions in 2D materials. (a–d) Calculated exciton dispersion curves for bilayer WSe_2_: (a) AA′ stacking at external electric field *E*_*z*_ = 0; (b) AB stacking at *E*_*z*_ = 0; (c) AA′ stacking at *E*_*z*_ = 2.056 V/nm; (d) AB stacking at *E*_*z*_ = 1.028 V/nm. Labels indicate direct and indirect excitons (DX and IDX) and layer localization nature: in-plane (IP), interlayer (IL), or hybrid interlayer (HIL). Adapted with permission from ref 91. Copyright 2025 the authors of ref 91, exclusive license to American Chemical Society. (e) Exciton dispersion of monolayer hBN. The π → π* (π → σ*) exciton branch is in green (red). The corresponding real-space exciton wave functions at different momenta are plotted in the insets. Adapted with permission from ref 17. Copyright 2023 American Physical Society. (f, g) Phonon-assisted luminescence in AA′-stacking bulk BN (top) and ABC-stacking rhombohedral bulk BN (bottom). Black: cathodoluminescence experiment. Green: *ab initio* exciton–phonon calculation. The phonon modes responsible for the peaks are labeled. Adapted with permission from ref 43. Copyright 2023 American Physical Society. (h) Phonon-assisted luminescence in cubic BN. Top: *T* = 10 K. Bottom: *T* = 300 K. Adapted with permission from ref 45. Copyright 2026 AIP Publishing.

Another example is provided by monolayer hexagonal boron nitride (hBN), whose exciton dispersion is shown in Figure 5e. Its low-energy excitonic landscape is dominated by two dispersion branches with distinct orbital character. As illustrated in the inset of Figure 5e, the green branch arises from electron–hole transitions between the π-like valence states localized on the nitrogen atom and the π* conduction states on the boron sublattice.^92^ In contrast, the red-colored branch stems from π → σ* electronic transitions, where the σ* electrons exhibit a nearly free character.^93^ Their relative ordering determines whether the excitonic ground state is momentum direct or indirect, thereby controlling the efficiency of phonon-assisted optical processes. While the nature of the band gap is crucial for spectroscopic probes like luminescence or EELS, determining it in monolayer hBN remains challenging. Experimentally, results are heavily influenced by substrate effects, whereas theoretically, predictions depend strongly on method-dependent and numerical choices such as pseudopotentials, lattice parameters, and quasiparticle corrections.

Once the excitonic landscape is established, exciton–phonon coupling dictates how these dark states gain optical activity providing interpretation to the observed photoluminescence spectrum.

In the case of layered BN, which is clearly an indirect system, exciton–phonon calculations permit to capture the phonon-assisted contribution to the luminescence spectra in terms of phonon replica peaks. These peaks originate from the recombination of finite-momentum dark excitons assisted by phonons supplying the required crystal momentum. Stacking provides an additional degree of control over the phonon-assisted spectra, as illustrated by the AA′ and ABC, or rhombohedral boron nitride (rBN), polytypes shown in Figure 5f,g.^43^ Both systems feature many clearly distinguishable phonon replicas due to transverse and longitudinal modes, both optical and acoustic. In rBN, however, the lack of horizontal mirror symmetry allows for the coupling of finite-momentum excitons with Z-phonon modes, i.e., the lattice vibrations with out-of-plane atomic oscillations. This coupling is forbidden in the AA′ case,^94^ where dark excitons may only radiatively recombine by absorbing or emitting phonon modes where the ions oscillate in the layer plane. As the two stackings have comparable stability and can both appear in epitaxially grown large BN sheets, this intrinsic spectroscopic fingerprint offers a robust route for identifying BN polytypes experimentally.

Temperature further reshapes indirect luminescence by thermally populating higher-energy dark excitons in addition to broadening the phonon replicas. In cubic boron nitride (cBN), the two lowest-lying excitons at **q** = **X** are separated by ∼5 meV, meaning that the higher-energy state becomes populated and can be activated at temperatures above 60 K. This is apparent in the intrinsic calculated PL spectrum, shown in Figure 5d.^45^ As a consequence, a new phonon-assisted emission peak emerges at room temperature, reflecting the thermal population of the second excitonic branch.

Beyond determining optical spectra, exciton–phonon coupling also influences carrier transfer in layered heterostructures, since intralayer excitons are strongly optically active, but interlayer excitons are the most relevant for device applications based on charge separation in the visible range. Phonon scattering can mediate the conversion between intra- and interlayer excitons through phonon-assisted interlayer hopping of electrons or holes, as proposed using TMD heterobilayers featuring a Janus layer providing an intrinsic electric field.^95^

Exciton–phonon scattering is also the main driver of nonequilibrium sub-band-gap dynamics in 2D semiconductors, strongly shaping phenomena such as coherent phonon generation,^96,97^ temperature-dependent carrier thermalization/exciton thermalization.^39^ Phonon-mediated dephasing and intervalley scattering limit coherence in ultrafast spectroscopy.^16^ In 2D quantum emitters, dephasing and phonon sidebands reduce single-photon indistinguishability, constraining quantum-information applications, although phonon-assisted processes can also drive near-unity excitation efficiency.^98^

Therefore, broadening the scope of the discussion, the extension of the *ab initio* BSE framework to include the effects of lattice vibrations represents an important quantitative tool for the engineering of optoelectronic properties in emerging 2D semiconductors (including temperature trends, scattering strengths, lifetimes, spectroscopic characterization, and nonequilibrium dynamics). As indirect excitonic systems become increasingly relevant for optoelectronic and photovoltaic applications, exciton–phonon coupling emerges as a fundamental design parameter rather than a secondary correction.

### Outlook: Design Rules for Excitonic and Anisotropic Optical Functionality

The emerging 2D semiconductors discussed in this MiniReview show how first-principles many-body calculations can connect microscopic excitonic physics to functional optical responses. Their behavior is determined by the combined influence of dielectric screening, crystal symmetry, orbital character, lattice vibrations, and interlayer electrostatics. These ingredients control exciton binding and localization, bright–dark ordering, polarization-dependent selection rules, phonon-assisted optical channels, and charge separation, thereby linking fundamental excitonic properties to applications ranging from polarization-sensitive photodetection to ultrathin solar-energy conversion.

Across these material families, distinct excitonic regimes emerge from the interplay of screening, symmetry, and dimensionality. Graphitic carbon nitrides and MoSi_2_N_4_ are characterized by low-energy dark excitons and comparatively long radiative lifetimes, favoring charge retention and energy conversion applications, whereas group-III nitrides combine strong exciton binding with bright ground states and ultrafast emission, making them more suitable for light-emitting functionalities. Low-symmetry systems such as SnO and tellurene instead display pronounced in-plane optical anisotropy and polarization-selective excitonic transitions, which are relevant for directional photodetection. Tellurene-based heterostructures add a further regime in which strong absorption is combined with type-II band alignment and spatial charge separation, targeting photovoltaic energy conversion.

Four complementary design principles emerge. First, the dielectric environment, controlled through substrates, encapsulation, layer number, or adjacent materials, provides a direct handle on quasiparticle and excitonic energy scales. Second, reduced symmetry, polymorphism, stacking, and anisotropic orbital character redistribute excitonic oscillator strength between crystallographic directions, enabling polarization-selective absorption and emission. Third, type-II band alignment and interlayer electrostatics in vdWHs spatially separate electrons and holes, promoting exciton dissociation and charge extraction while preserving strong light absorption. Finally, exciton–phonon coupling governs relaxation between bright and dark states and activates momentum-forbidden optical transitions, making temperature and lattice dynamics integral components of the optical response rather than secondary sources of broadening.

The experimental validation of these design rules requires complementary probes capable of resolving the relevant excitonic states and their dynamics. Temperature-dependent photoluminescence and absorption can identify thermally activated excitons, phonon replicas, and energy separation between bright and dark states. Polarization-resolved absorption, emission, and Raman spectroscopy can directly test symmetry-controlled selection rules and anisotropic orbital character. Time-resolved measurements are essential for distinguishing exciton formation, intervalley relaxation, radiative decay, and interlayer transfer. Direct comparison with *GW*-BSE and exciton–phonon calculations will be particularly valuable for assigning spectral features and determining which microscopic mechanism dominates the measured response.

An important future direction is the simultaneous optimization of properties that are often treated separately. Strong exciton binding can stabilize optical excitations but hinder charge separation, spatially indirect excitons promote long lifetimes and extraction but reduce oscillator strength, and pronounced optical anisotropy does not necessarily coincide with broadband absorption. The most promising materials and heterostructures will therefore be those in which these competing requirements can be balanced through composition, polymorphism, stacking, and the dielectric environment. First-principles many-body calculations can play a decisive role in identifying such trade-offs before synthesis and in establishing whether a proposed material is better suited for light emission, polarization-sensitive detection, or solar energy conversion.

Looking further ahead, moiré superlattices and engineered dielectric environments offer additional degrees of freedom to control exciton localization, interactions, and optical selection rules.^99^ In parallel, machine-learning approaches can accelerate first-principles materials discovery by learning expensive many-body corrections from lower-cost calculations. For example, *GW* quasiparticle band structures of 2D materials have been accurately reconstructed from DFT-derived descriptors,^100^ opening a route toward large-scale screening of materials with targeted excitonic and optical properties.

The central challenge is to identify which combination of screening, symmetry, lattice dynamics, and interfacial alignment yields the desired optical functions. Combining predictive many-body theory with targeted spectroscopy provides a route to rationally selecting materials for anisotropic photodetection, light emission, and ultrathin solar-energy conversion.
